# Altered Memory Circulating T Follicular Helper-B Cell Interaction in Early Acute HIV Infection

**DOI:** 10.1371/journal.ppat.1005777

**Published:** 2016-07-27

**Authors:** Roshell Muir, Talibah Metcalf, Virginie Tardif, Hiroshi Takata, Nittaya Phanuphak, Eugene Kroon, Donn J. Colby, Rapee Trichavaroj, Victor Valcour, Merlin L. Robb, Nelson L. Michael, Jintanat Ananworanich, Lydie Trautmann, Elias K. Haddad, RV254/SEARCH010 RV304/SEARCH 013 Study Groups

**Affiliations:** 1 Drexel University, Department of Medicine, Division of Infectious Diseases & HIV Medicine, Philadelphia, Pennsylvania, United States of America; 2 The Henry M. Jackson Foundation for the Advancement of Military Medicine, Bethesda, Maryland, United States of America; 3 United States Military HIV Research Program, Bethesda, Maryland, United States of America; 4 SEARCH, the Thai Red Cross AIDS Research Centre, Bangkok, Thailand; 5 Department of Retrovirology, Armed Forces Research Institute of Medical Sciences, United States Component, Bangkok, Thailand; 6 Memory and Aging Center, University of California, San Francisco, United States of America; Vaccine Research Center, UNITED STATES

## Abstract

The RV254 cohort of HIV-infected very early acute (4thG stage 1 and 2) (stage 1/2) and late acute (4thG stage 3) (stage 3) individuals was used to study T helper- B cell responses in acute HIV infection and the impact of early antiretroviral treatment (ART) on T and B cell function. To investigate this, the function of circulating T follicular helper cells (cTfh) from this cohort was examined, and cTfh and memory B cell populations were phenotyped. Impaired cTfh cell function was observed in individuals treated in stage 3 when compared to stage 1/2. The cTfh/B cell cocultures showed lower B cell survival and IgG secretion at stage 3 compared to stage 1/2. This coincided with lower IL-10 and increased RANTES and TNF-α suggesting a role for inflammation in altering cTfh and B cell responses. Elevated plasma viral load in stage 3 was found to correlate with decreased cTfh-mediated B cell IgG production indicating a role for increased viremia in cTfh impairment and dysfunctional humoral response. Phenotypic perturbations were also evident in the mature B cell compartment, most notably a decrease in resting memory B cells in stage 3 compared to stage 1/2, coinciding with higher viremia. Our coculture assay also suggested that intrinsic memory B cell defects could contribute to the impaired response despite at a lower level. Overall, cTfh-mediated B cell responses are significantly altered in stage 3 compared to stage 1/2, coinciding with increased inflammation and a reduction in memory B cells. These data suggest that early ART for acutely HIV infected individuals could prevent immune dysregulation while preserving cTfh function and B cell memory.

## Author Summary

The HIV-specific T cell memory response diminishes rapidly even after the initiation of anti-retroviral treatment (ART), and there is no control of viral rebound if treatment is interrupted. Restoration or preservation of memory T cells or B cells with treatment, to allow for control of virus replication after ART is stopped, is therefore very important. CD4^+^ T cells, in particular T follicular helper (Tfh) cells, have a major role in mediating antiviral immunity by providing help to B cells, which is key to a strong and efficient anti-HIV antibody response. The unique RV254 cohort provided the best setting to analyze immune responses during very early acute HIV, as the study was able to enroll individuals that were infected for less than 2 weeks and initiated treatment approximately 1–2 days after recruitment. We aimed to study the capacity of memory circulating Tfh (cTfh) cells to promote B cell help in acute HIV infection, and found the interaction to be dysfunctional in the later stage compared to the very early stages, accompanied by increased levels of proinflammatory cytokines and a reduction in regulatory cytokines. High levels of plasma viremia correlated with low cTfh-mediated B cell antibody production in later stage acute individuals; and memory B cells were significantly decreased but could be restored with ART, compared to chronically infected individuals, who could not normalize this compartment compared to healthy individuals. Overall, we show that the cTfh- B cell interaction and B cell memory compartment is altered in late stage acute infection, mainly attributed to an increase in inflammation and skewing of the response away from helper to proinflammatory. Identifying individuals for treatment in the earliest stages of acute infection, prior to immune damage, could preserve cTfh function and the anti-HIV B cell response, therefore reducing the chances of viral rebound upon the cessation of ART.

## Introduction

The progressive nature of immune dysfunction in HIV infected individuals has implied that early treatment could play a critical role in reducing immune defects and in preserving T and B cell memory responses against HIV infection [1–3]. Despite the fact that antiretroviral treatment (ART) has been pivotal in reducing the viral burden in persons infected with HIV, the concurrent decline in the HIV-specific T and B cell memory response poses a great risk, as treatment interruption can lead to a loss in the control of viremia [4–7]. The need to identify immune parameters that are associated with preservation of the memory response during HIV infection is therefore important to providing clues for therapies going forward.

Even with ART, low level HIV replication in lymphoid tissues has been shown to maintain a state of chronic immune activation [8]. B cell hyperactivation, a hallmark feature of HIV infection, is characteristically evidenced by elevated serum immunoglobulin [9, 10], and also includes changes within the circulating B cell compartment, some of which cannot be reversed by ART as evident in chronic individuals [11]. These changes include an increase in their activation, proliferation, immature and terminal differentiation markers [12–16], as well as a reduction in CD27^+^ memory cells [17–19].

CD4 T follicular helper cells (Tfh) are specialized in providing help to B cells and support B cell maturation and differentiation to long-lived plasma cells in the germinal center (GC) [20]. It therefore follows that if an efficient HIV-specific B cell response is to be achieved, Tfh function must be preserved. With access to human lymphoid tissue limited, there has been an increased interest in the study of CD4^+^CXCR5^+^ T cells in the blood known as memory circulating Tfh (cTfh), that are also very efficient at inducing B cell differentiation and providing B cell help; and much progress has been made to characterize and understand their biology [6, 21–23]. We have previously shown that germinal center Tfh cells in HIV positive lymph nodes are dysregulated and that the B cell response is severely reduced compared to those from HIV negative lymph nodes [24]. We have also recently shown that HIV-associated microenvironment can affect the differentiation and phenotype of cTfh cells, and that these cells from chronic aviremic individuals treated in very late stages (3–6 months after transmission) are dysfunctional in providing B cell help compared to elite controllers or healthy individuals [6]. It is therefore plausible that individuals who undergo very early ART could preserve their CD4^+^ Tfh function, and that phenotypic perturbations of T and B cell populations that are characteristic of acute HIV infection are better resolved in very early treated individuals. As the HIV-associated microenvironment likely affects the Tfh program [6], it is also possible that in very early acute HIV-infected individuals, there is less immune activation and therefore fewer inflammatory signals, which would reduce the risk for adverse effects on Tfh phenotype and function.

The unique RV254/SEARCH010 Thai cohort is strategic in that HIV infected participants are recruited during the earliest stage of acute infection and receive ART almost immediately [25, 26]. This population of individuals offers the best setting to accurately analyze the benefits of early ART on immune preservation and function. Using cTfh cells as surrogate B-cell helper T cells, we examined T-helper mediated function in stage 1/2 (4^th^G stages 1 and 2 grouped) (early acute; median average 12 and 17 days respectively from history of exposure) and stage 3 (4^th^G stage 3) (late acute; median average 18 days from history of exposure) [27]; and investigated the phenotype and immune activation status of cTfh and memory B cells before and post ART. We hypothesized that cTfh-mediated B cell help in untreated late acute individuals will be reduced compared to very early acute individuals and lead to an increased proinflammatory environment.

## Results

### Memory cTfh- mediated B cell help is impaired in late stage acute HIV-infected individuals

We initially examined whether the frequency and phenotype of cTfh cells (CXCR5^+^CXCR3^-^) in peripheral blood from stage 1/2 are similar to those from stage 3 at week 0. Similar frequencies of cTfh cells were observed in all stages (P = 0.62, S1A and S1B Fig). In addition, there was no significant difference in the expression of inhibitory (PD-1) (P = 0.65), costimulatory (ICOS) (P = 0.85) or activation markers (CD25, CD38) (P = 0.27 and P = 0.82 respectively) when we compared stage 1/2 to stage 3 (S1C–S1F Fig). These results suggest that there are no apparent differences in the phenotype of cTfh cells between the two groups. Additionally we did not observe any differences in the induction of CD38 expression on cTfh cells (P = 0.55) between the two stages after SEB stimulation (S1G Fig). Thus we studied the function of cTfh cells between the two groups in the context of interaction with memory B cells.

**Fig 1:**
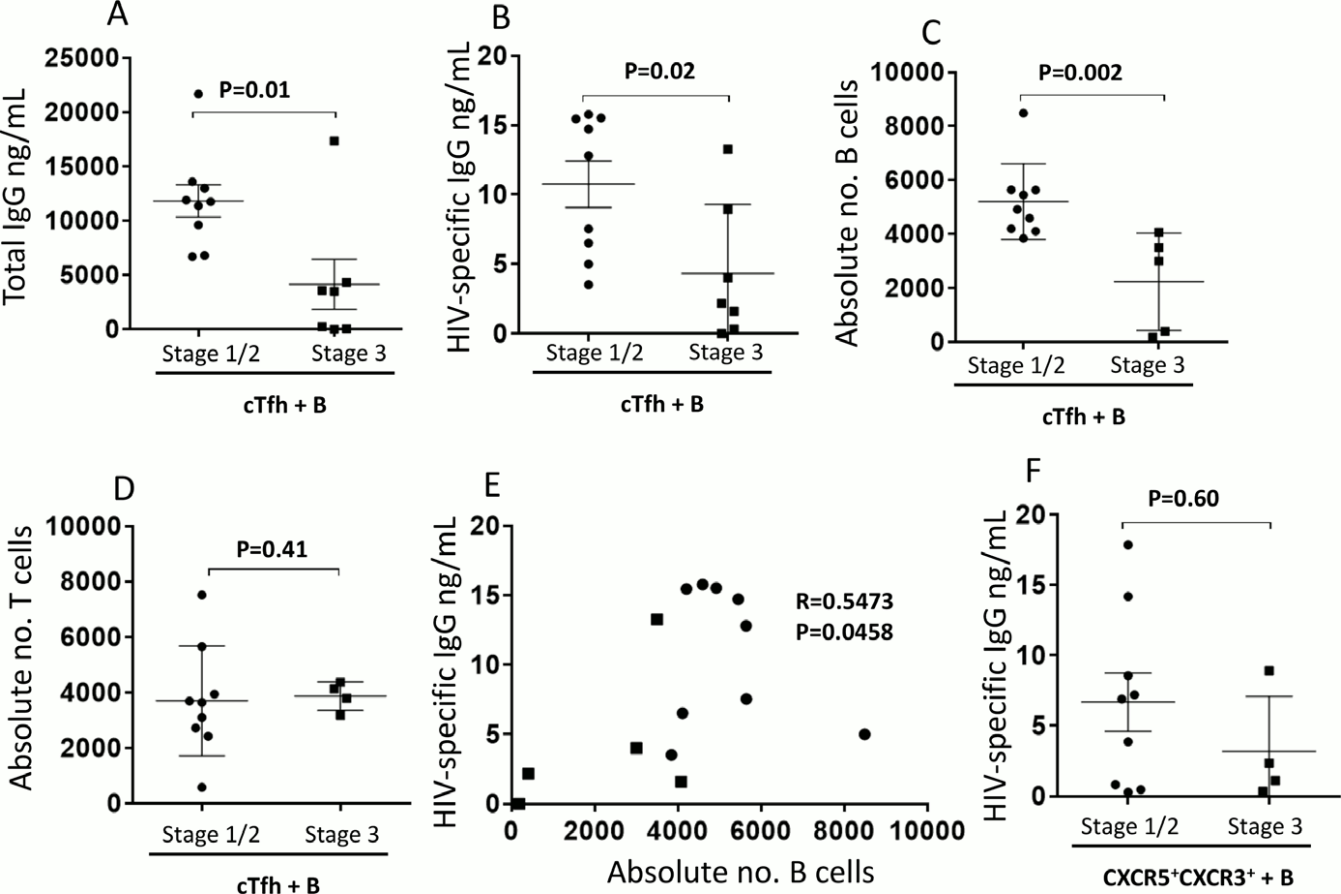
cTfh-mediated memory B cell help is impaired in late stage acute HIV-infected individuals. PBMCs from week 0 stage 1/2 (n = 9) and stage 3 (n = 4–7) individuals were sorted. cTfh (CD3^+^CD4^+^CD45RA^-^CXCR5^+^CXCR3^-^) or the less efficient T-helper cells (CD3^+^CD4^+^CD45RA^-^CXCR5^+^CXCR3^+^) denoted CXCR5^+^CXCR3^+^ were placed in culture with autologous resting memory B cells (CD19^+^CD10^-^CD21^+^CD27^+^) in the presence of SEB. Quantification of cTfh-mediated B cell help was carried out by measuring (A) total and (B) HIV-specific IgG ELISA in 7 day culture supernatant. Absolute numbers of live (C) B and (D) T cells were quantified. (E) Correlation analysis was carried out between the absolute number of B cells and HIV-specific IgG. (F) HIV-specific IgG ELISA in 7 day culture supernatant from cultures of CXCR5^+^CXCR3^+^ T cells and memory B cells was also quantified. For (A)-(D) and (F) bars represent mean ±SD. Symbols on the graphs represent stage 1/2 individuals (black circles) and stage 3 individuals (black squares) and statistics were carried out using the Mann-Whitney non-parametric test. For (E) symbols represent stage 1/2 individuals (black circles) and stage 3 individuals (black squares) and correlation data was analyzed using Spearman r test. * P< 0.05. Results were significant if P < 0.05.

To investigate cTfh function at different stages very early in acute infection, coculture assays of sorted cTfh cells and autologous-sorted resting memory B cells (CD21^+^CD27^+^) were performed. Cocultures of cTfh cells from stage 3 elicited significantly less help to B cells compared to cocultures of cTfh cells from stage 1/2 individuals, as evidenced by reduced levels of total IgG (P = 0.01, Fig 1A), HIV-specific IgG (P = 0.02, Fig 1B); and reduced number of absolute B cells in the coculture at day 7 (P = 0.002, Fig 1C). Similar numbers of cTfh cells survived through the end of the 7-day coculture in both stage 1/2 and stage 3 (P = 0.41, Fig 1D). The data indicated that the blunted antibody response in the coculture of stage 3 was independent of T cell survival and points to a defective T/B cell interaction. Nonetheless, these results signify preservation of the cTfh- dependent B cell and HIV- specific IgG response in the earliest acute stages of HIV infection compared to the later stages. This is further demonstrated by a positive correlation between absolute B cell numbers and HIV-specific IgG production in coculture (P = 0.046, R = 0.5473; Fig 1E). There were no significant differences observed in HIV-specific IgG (P = 0.60, Fig 1F) production from cocultures of less efficient B-helper T cells (CXCR5^+^CXCR3^+^) suggesting that the defect in stage 3 is specific for cTfh/B cell interaction. Of note, although CXCR5^+^CXCR3^-^ cTfh cells are mostly enriched in a subset that provides efficient B cell help, the CXCR5^+^CXCR3^+^ T cell subset can also provide help, but to a lesser degree (S2 Fig), hence they are a more appropriate control population compared to CXCR5^-^ non-cTfh cells.

### IL-10 and TNF-α influence cTfh-mediated HIV-specific IgG production

**Fig 2:**
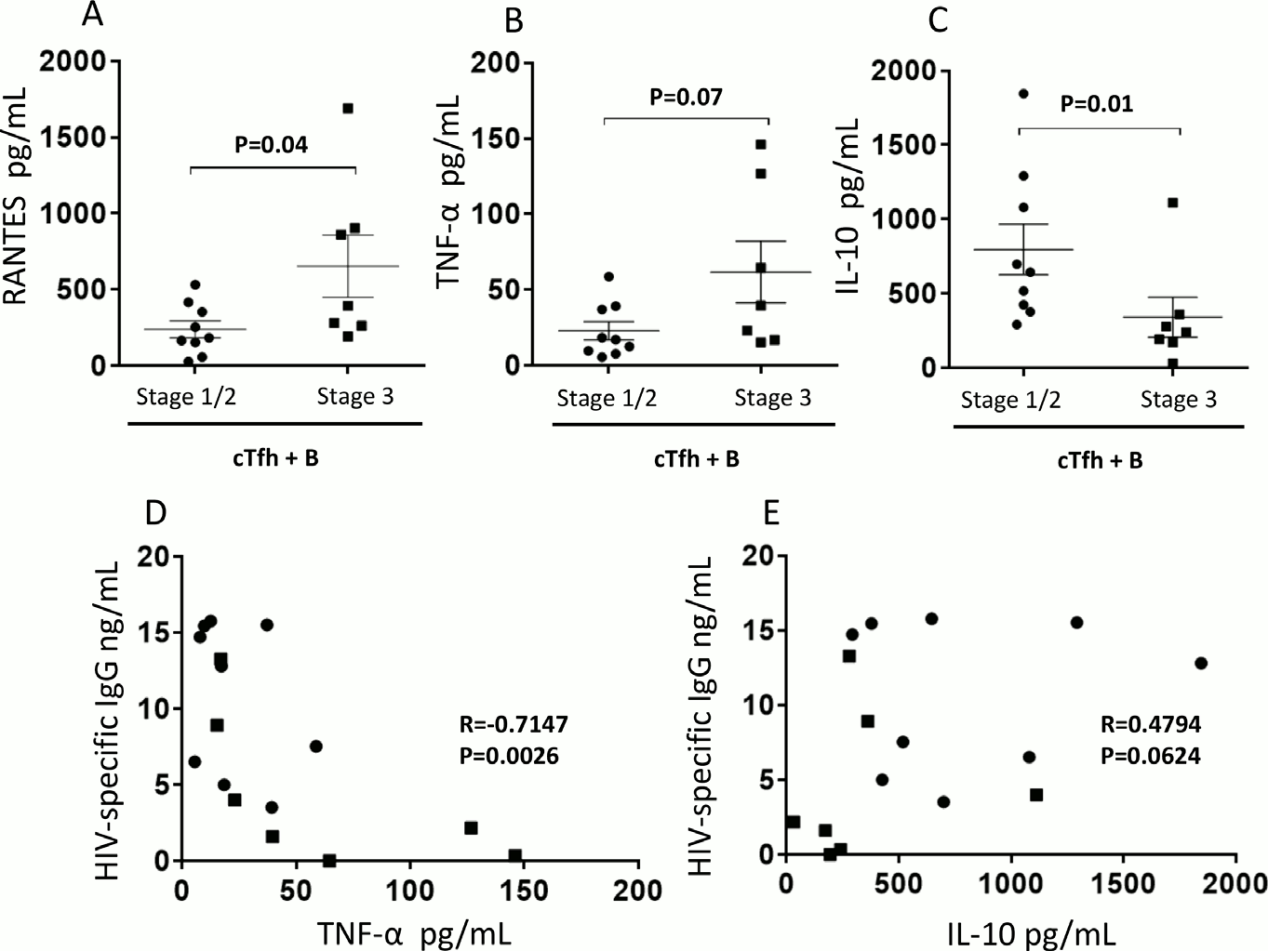
IL-10 and TNF-α levels in cocultures of cTfh and B cells from late stage acute individuals influence HIV-specific IgG production. Cocultures of cTfh and autologous resting memory B cells from week 0 stage 1/2 (n = 9) and stage 3 (n = 7) individuals were analyzed for the presence of cytokines including (A) RANTES, (B) TNF-α and (C) IL-10. Correlation analysis was carried out between (D) TNF-α; (E) IL-10 and HIV-specific IgG. For (A)-(C) bars represent mean ±SD. Symbols on the graphs represent stage 1/2 individuals (black circles) and Stage 3 individuals (black squares) and statistics were carried out using the Mann-Whitney non-parametric test. For (D) and (E) symbols represent stage 1/2 individuals (black circles) and stage 3 individuals (black squares) and correlation data was analyzed using Spearman r test. * P< 0.05.

To evaluate whether our observations on reduced B cell responses in stage 3 cocultures may be related to a skewed cTfh program, we analyzed cocultures of cTfh cells from stage 1/2 and 3 for cytokine production. In the cTfh cocultures of stage 3 individuals, there was a significant increase in the Th1 cell chemotactic cytokine RANTES [28] (P = 0.04, Fig 2A) and a trend towards an increase in the acute phase proinflammatory cytokine TNF-α [29] (P = 0.07, Fig 2B), compared to stage 1/2 individuals. Concurrently, levels of the regulatory cytokine IL-10 [30] were significantly reduced in stage 3 individuals when compared to those of stage 1/2 (P = 0.01 Fig 2C). We also observed an apparent increase in levels of the proinflammatory cytokines IL-1β (P = 0.299), IL-6 (P = 0.142), IFN-γ (P = 0.110) and MIP-1α (P = 0.343) in stage 3 compared to stage 1/2, although this did not reach statistical significance (S3A–S3D Fig). Importantly, we observed a negative correlation between TNF-α and HIV-specific antibody levels in the coculture supernatants (P = 0.003, R = -0.715; Fig 2D) suggesting a negative impact of TNF-α on cTfh/B cell interaction. On the other hand, we observed a trend towards a positive correlation between IL-10 and HIV specific antibody levels in the coculture supernatants (P = 0.062, R = 0.480; Fig 2E) suggesting a positive impact of IL-10 on cTfh/B cell help. This is not surprising as IL-10 is a known potent growth and differentiation factor for B cells and can be secreted by Tfh cells [31, 32]. Overall, these results support the notion that delayed ART could cause alterations in cTfh program towards a Th1 profile as in stage 3 which could affect cTfh-dependent antibody output and phenotype.

### Elevated plasma viral load is associated with Tfh impairment and humoral immune defects

**Fig 3:**
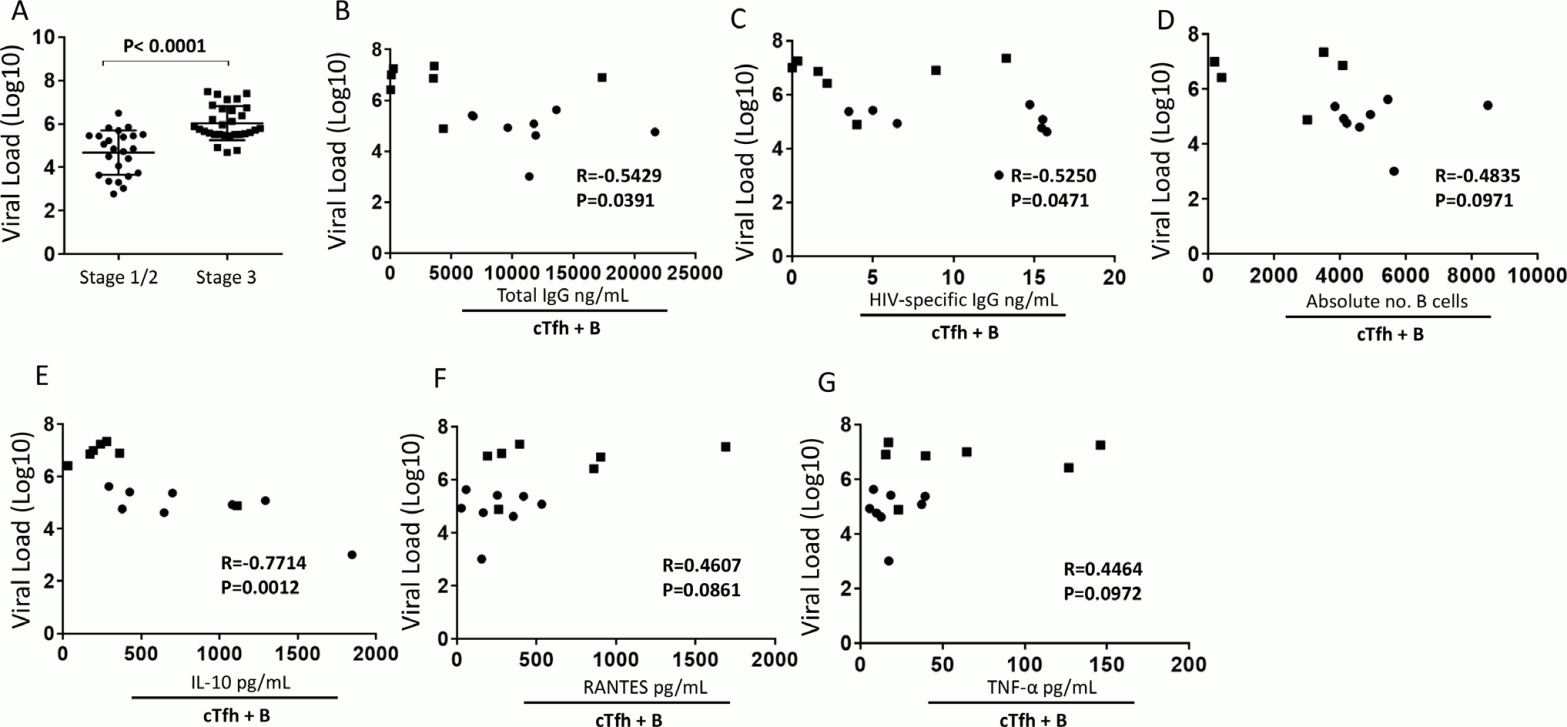
Elevated plasma viral load is associated with reduction in the cTfh-dependent humoral response. (A) Viral load was quantified in the plasma from stage 1/2 (n = 24) and from stage 3 (n = 30) HIV-infected individuals at day 0 prior to ART initiation. Correlation analysis was carried out between plasma viral load at day 0 and cTfh and memory 7 day culture supernatant (B) -Total IgG, (C) -HIV-specific IgG, (D) absolute number of B cells, (E) IL-10, (F) RANTES and (G) TNF-α from stage 1/2 (n = 8) and stage 3 (n = 5–7). For (A) bars represent mean SD, symbols on the graphs represent stage 1/2 individuals (black circles) and stage 3 individuals (black squares) and statistics were carried out using the Mann-Whitney non-parametric test. For (B)-(G) symbols represent stage 1/2 individuals (black circles) and stage 3 individuals (black squares) and correlation data was analyzed using Spearman r test.* P < 0.05.

**Fig 4:**
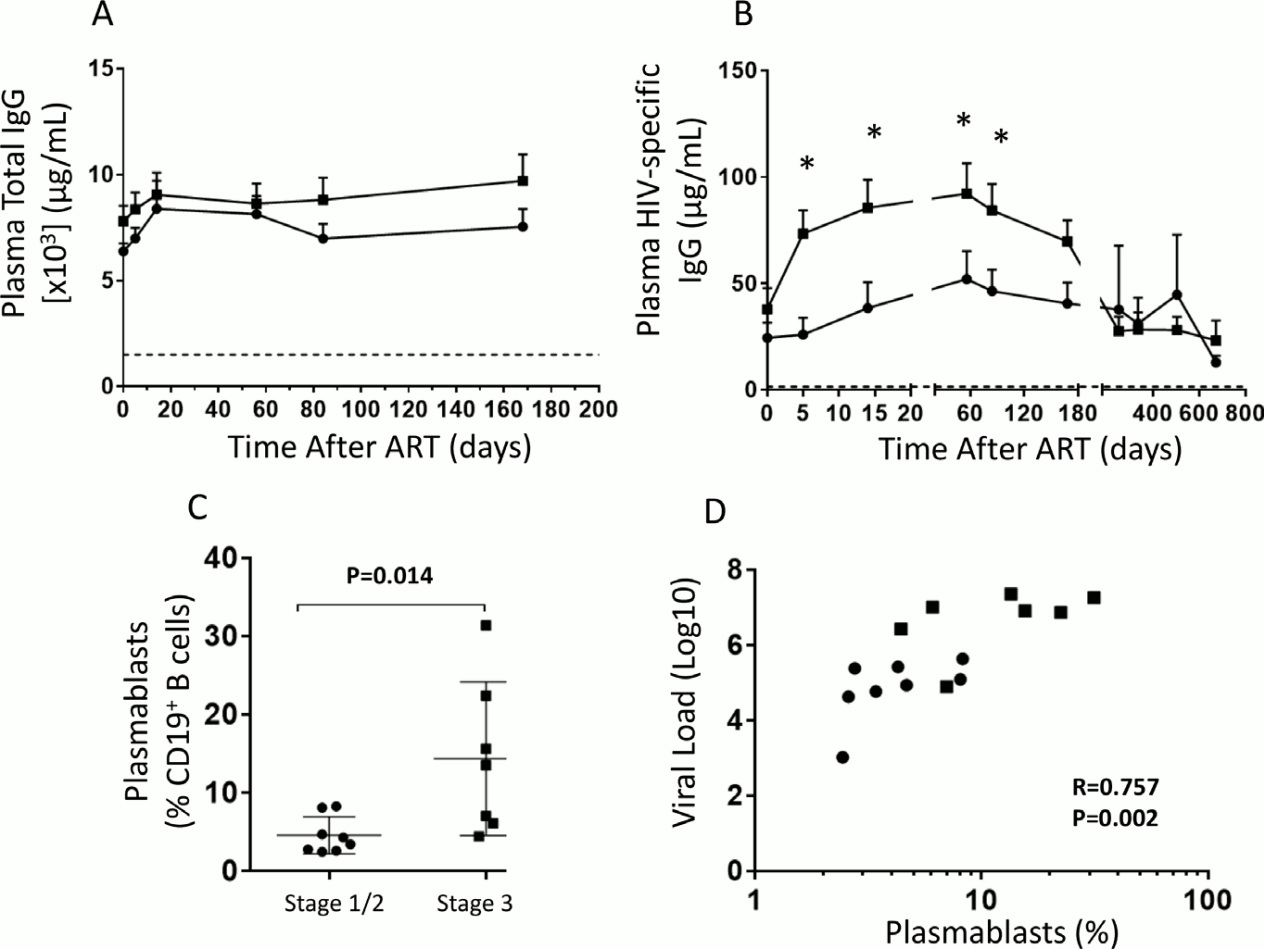
Elevated plasma viral load is also associated with a T cell-independent hyperactivated B cell response. Plasma at day 0 and days 5, 14, 56, 84, 168, 252, 336, 504 and 672 of ART, were analyzed by ELISA for (A) Total IgG and (B) HIV-specific IgG. PBMCs from week 0 Stage 1 and 2 (n = 8) and Stage 3 (n = 7) patients were analyzed by flow cytometry for terminally differentiated plasmablasts identified as CD19^+^CD10^-^CD20^-^CD21^-^CD38^hi^CD27^hi^ cells. (C) Plasmablasts from stage 1/2 and stage 3 were compared to each other and (D) together with plasma viral load. For graphs (A) and (B) bars represent mean SEM and statistics were carried out using multiple T tests comparing stage 1/2 to stage 3 at the different time points. Symbols on the graphs represent Stage 1/2 individuals (black circles) and Stage 3 individuals (black squares). For (C) bars represent mean ±SD, symbols on the graphs represent stage 1/2 individuals (black circles) and stage 3 individuals (black squares) and statistics carried out using the Mann-Whitney non-parametric test. For (D) symbols represent stage 1/2 individuals (black circles) and stage 3 individuals (black squares) and correlation data was analyzed using Spearman r test. * P < 0.05.

We found that plasma viral load was significantly lower in stage 1/2 compared to stage 3 (P< 0.0001, Fig 3A). This suggested that elevated levels of plasma viral load could be associated with cTfh cell mediated B cell defects. To ascertain this association, we measured the correlation between plasma viral load in both stages and antibody levels in the cocultures of cTfh and memory B cells. Interestingly, we found a significant negative correlation between plasma viral load and total IgG in the coculture supernatants (P = 0.039, R = -0.543; Fig 3B). This data provides evidence that elevated viral load can skew cTfh function towards a non-helper program. Similarly, we observed a significant negative correlation between plasma viral load and HIV-specific IgG antibody levels (P = 0.047, R = -0.525; Fig 3C). As expected, a negative correlation trend was observed between plasma viral load and absolute number of B cells in the coculture (P = 0.097, R = -0.484; Fig 3D). Importantly we found a significant negative correlation between viral load and IL-10 concentration in the supernatants of the coculture assay (P = 0.001, Fig 4E). On the other hand, a trend towards a positive correlation was observed between viral load and RANTES (P = 0.086, R = 0.461; Fig 3F) and TNF-α (P = 0.097, R = 0.446; Fig 3G) supernatant levels. Overall these results indicate that prolonged plasma viral load, as in seen in stage 3, could be responsible for blunting cTfh mediated B cell help.

### Elevated plasma viral load is also associated with a virus-induced hyperactivated antibody response

We determined whether elevated plasma viral load in stage 3 was associated with a hyperactivated antibody response and altered B cell differentiation. Although individuals in both groups had similar levels of plasma total IgG from the time of ART initiation and throughout treatment (Fig 4A), stage 1/2 individuals had significantly less HIV-specific IgG from as early as day 5 of ART compared to stage 3 (P = 0.003, Fig 4B), indicating increased B cell hyperactivation in late acute infection that could be partly due to antigen overload. It is possible that this hyper activation of B cells occurs in extrafollicular areas as damage to lymph nodes occurs very early in acute infection as has been previously shown [33]. This could contribute to hypergammaglobulinemia which could directly affect cTfh/B cell interactions as we observed in stage 3 (Fig 1A and 1B). In the HIV viremic setting, plasmablasts are markers of uncontrolled proliferation and exhaustion and have been shown phenotypically and functionally to be increased and to secrete antibodies *ex vivo* [9, 13, 34]. Frequencies of plasmablasts were significantly increased in stage 3 individuals prior to ART initiation, compared to stage 1/2 individuals (P = 0.014, Fig 4C and S4A Fig); and that this increase in percentages of plasmablasts correlate positively with an increase in viral production (P = 0.002, R = 0.757, Fig 4D). This suggests that the enhanced frequency of plasmablasts and the HIV-specific antibody response, could be in part due to the enhanced inflammatory microenvironment at stage 3 when compared to stage 1/2.

### Alterations in circulating mature B cell subpopulations in acute HIV infection

**Fig 5:**
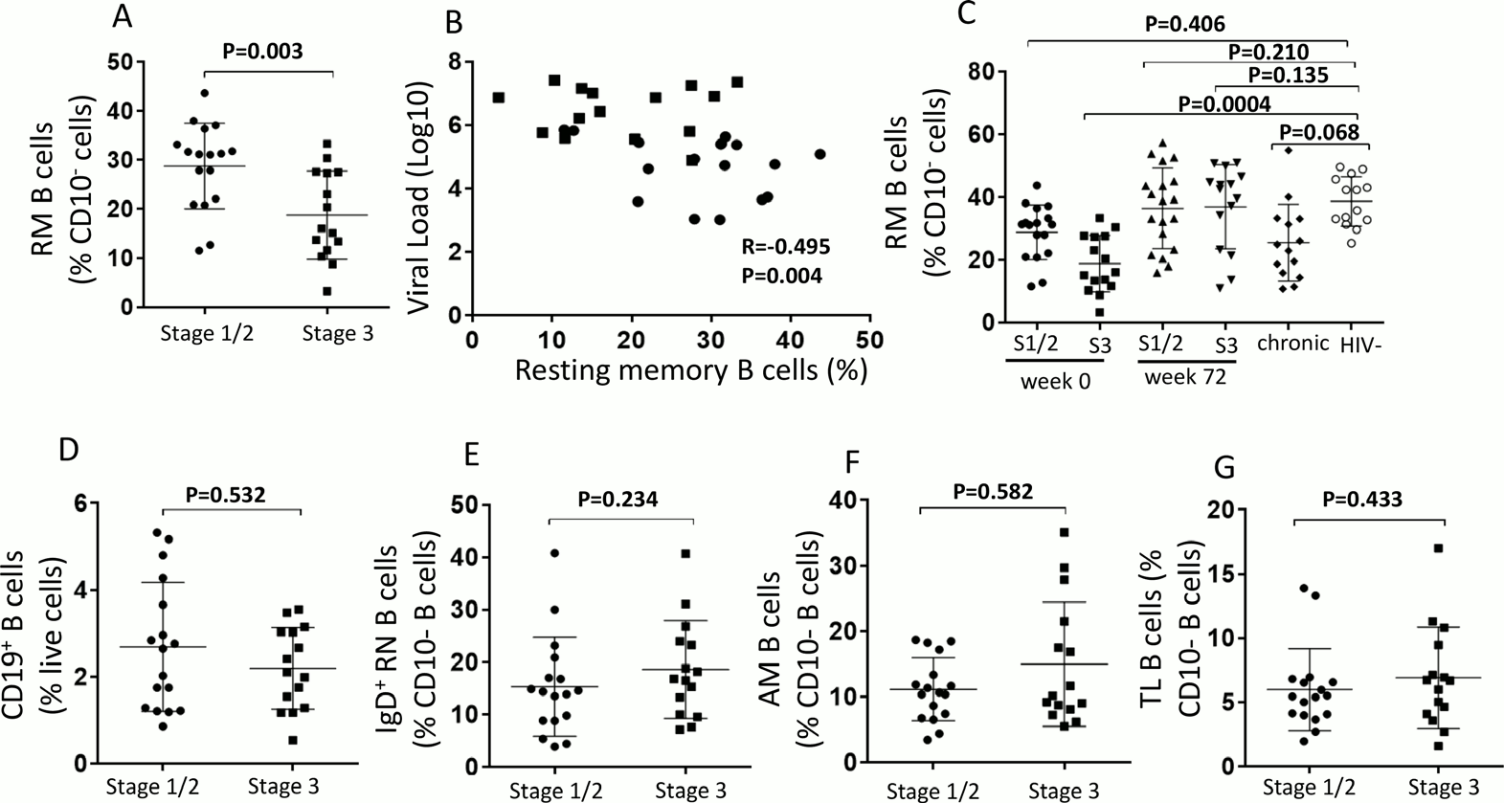
Impact of early acute HIV infection on blood memory B cell subsets. PBMCs from week 0 stage 1/2 (n = 17) and stage 3 (n = 16) patients were analyzed by flow cytometry for resting memory (RM) B cells CD19^+^CD10^-^CD21^+^CD27^+^ and (A) compared with each other and (B) with plasma viral load. (C) Comparisons were also done with PBMCs from week 72 of ART (W72) (n = 19 and n = 18 respectively), chronic HIV infected patients (n = 14) and HIV negative patients (n = 14). PBMCs from week 0 stage 1/2 (n = 17) and stage 3 (n = 16) patients were analyzed by flow cytometry for (D) Total B cells which were identified as CD19^+^; (E) resting naïve (RN) B cells CD19^+^CD10^-^CD21^+^CD27^-^IgD^+^ (F) activated memory (AM) B cells CD19^+^CD10^-^CD21^-^CD27^+^ cells, (G) Tissue-like (TL) B cells CD19^+^CD10^-^CD21^-^CD27^-^. For (A) and (E)-(H), bars represent mean ±SD. Symbols on the graphs represent stage 1/2 individuals (black circles) and stage 3 individuals (black squares). Statistics were carried out using the Mann-Whitney non-parametric test. For (B) symbols represent stage 1/2 individuals (black circles) and stage 3 individuals (black squares) and correlation data was analyzed using Spearman r test. * P < 0.05. For (C), symbols represent individuals from week 0 stage 1/2 (black circles), week 0 stage 3 (black squares), week 72 ART stage 1/2 (upward pointing black triangles), week 72 ART stage 3 (downward pointing black triangles), chronic individuals (black diamonds) and HIV negative (open circles). Statistics were carried out using Kruskal-Wallis multiple comparison test. * P< 0.05.

HIV infection leads to early and progressive depletion of peripheral CD27^+^ resting memory B cells [11, 18]. This loss occurs from the onset of acute infection [33, 35] and persists even after prolonged ART [11]. We studied whether resting memory B cells (S4B Fig) are affected at different stages of acute HIV infection. We observed lower frequencies of resting memory B cells in stage 3 individuals compared to stage 1/2 (P = 0.003, Fig 5A), and this decrease correlated with an increase in plasma viral load (P = 0.004, R = -0.495, Fig 5B).

Importantly, administration of ART at stage 1/2 and at stage 3 preserved the frequency of resting memory B cells (similar frequencies to HIV negative subjects) (stage 1/2, P = 0.210 and stage 3, P = 0.135; Fig 5C). Whereas, initiation of ART much later at the chronic stage (months after transmission), was not able to adequately restore the resting memory B cell compartment (p = 0.068, Fig 5C). It is worth noting that the frequency of resting memory B cells at week 0 of stage 1/2 is similar to that of healthy individuals (P = 0.406); however the frequencies at week 0 of stage 3 are significantly reduced compared to healthy subjects (P = 0.0004) and is similar to that from chronic (P>0.999). In addition, we did not see significant differences between the stages, prior to ART, in the frequency of total CD19^+^ B cells, IgD^+^ resting naïve B cells (CD21^+^CD27^-^), activated memory B cells (CD21^-^CD27^+^) or exhausted tissue-like B cells (CD21^-^CD27^-^) between stage 1/2 and stage 3 (Fig 5D–5G).

## Discussion

In this study, we found that the cTfh-B cell interaction is significantly altered in later stage acute HIV infection. Supernatants from these cocultures showed that total and HIV-specific IgG from stage 3 individuals not yet on ART are significantly reduced compared to stage 1/2 individuals. We further found that the reduced levels of HIV-specific antibodies coincided with reduced B cell numbers in the coculture supernatants. Our previously published reports have demonstrated that the HIV microenvironment can negatively impact Tfh mediated B cell help [6, 24]. Specifically, we showed cTfh cells obtained from HIV infected individuals who were treated relatively late (months after transmission) displayed a Tfh1 inflammatory phenotype that is not capable of providing help to B cells. We therefore sought to investigate how early this impairment occurs and examine the underlying mechanisms using the unique RV254 cohort of acute HIV-infected subjects. This work has showed that cTfh cells are impaired in their ability to provide B cell help as early as stage 3 (~18 days) of acute HIV infection. The reduction in absolute numbers of B cells after coculture of cells from stage 3 individuals correlated with increased HV-specific IgG secretion, which provides further evidence that the B cell response, as evidenced by antibody production, is directly linked to cTfh-mediated help. These results indicate that the cTfh-mediated B cells response is better preserved at the earliest stage of HIV infection and that identifying individuals very early on during HIV infection has the potential to preserve cTfh-dependent B cell responses.

To further characterize the dysregulation in cTfh-B cell interaction, we performed a coculture assay where we replaced cTfh cells from stage 3 (defective) with cTfh from healthy individuals (effective) in coculture with memory B cells (obtained from stage 3). We found that replacing cTfh showed signs of increased total and HIV-specific antibody levels (S5A and S5B Fig), as well as increased IL-10 and reduced RANTES levels (S5C and S5D Fig). This does suggest that reducing inflammation and hence reversing impairment of the cTfh-mediated B cell response can potentially be achieved by normalization of cTfh function.

In order to further discern the contribution of memory B cells and determine the extent of B cell impairment prior to coculture with cTfh, we measured memory B cell proliferation and antibody production following polyclonal stimulation with CpG oligonucleotide. Our results indicated that similar proliferation profiles between the two stages and apparent non-significant increase in total IgG production in stage 1/2 when compared to stage 3 (S6A and S6B Fig). Overall, this suggested some level of intrinsic memory B cell defect that could contribute to impaired antibody response in stage 3. Of note, we observed similar expression of the survival molecule Bcl-2 on memory B cells in both stages (S6C Fig). Overall our results suggest that the impairment of the cTfh-B cell interaction is primarily due to impaired interaction of cTfh and memory B cells, however we cannot overlook the contribution of intrinsic memory B cell defect to this phenomenon.

Additionally, we found that cocultures of cTfh cells obtained from patients at stage 3 are polarized towards a proinflammatory phenotype. Our results imply that this skewing of cTfh cells towards a non-cTfh program is associated with elevated levels of viral load observed at stage 3 when compared to stage 1/2. This has been confirmed by the significant correlation between viral load at week 0 and the impaired cTfh function as measured by the production of total and HIV-specific antibodies in culture supernatants. It is not clear from our study, however, whether this impairment is a direct effect of the virus or indirectly through viral-induced microenvironmental changes. Previously published data has suggested elevated proinflammatory cytokine levels including RANTES and TNF-α during chronic HIV infection [28, 29]. This is corroborated by findings from our own studies showing that cTfh from chronic individuals can be polarized to a more Th1 profile through the production of IL-2, TNF-α and IFN-γ [6]. cTfh cells can be further phenotyped into Tfh1, Tfh2 and Tfh17 subsets that provide help to B cells at varying levels and are characterized depending on their expression of CXCR3 and CCR6 [21, 36–38]. The impact that proinflammatory cytokines have on cTfh polarization in this setting would be of great interest.

The regulatory cytokine IL-10 has been shown to be important in B cell proliferation and maintenance [30–32, 39–41], as well as promoting Tfh-dependent responses in HIV and in other disease models [23, 24, 42]. In fact, lack of IL-10 production by CD4^+^ T helper cells in X-linked lymphoproliferative disease (XLP) patients impaired the development of B cell responses [43]. The increase in IL-10 and decreased proinflammatory cytokines, as well as the positive correlation between IL-10 and coculture HIV-specific IgG we observed in stage 1/2 individuals compared to stage 3, suggests that IL-10 could not only be important in regulating the early acute cytokine environment during HIV infection, but also in promoting cTfh-dependent B cell responses.

Rapid initiation of ART for maintaining low viremia and preserving immunity is a key objective of the RV254 study. We determined that stage 1/2 individuals had significantly lower viral load than stage 3 and consequently less plasma HIV-specific IgG and terminally differentiated B cells compared to stage 3 individuals. We know one of the characteristic effects of increased viremia on the B cell response is hypergammaglobulinemia [9, 10]. In addition, it has not been fully established whether cTfh interactions with memory B cells represent exactly what happens in the follicular microenvironment. However, based on our results, it could be that the hypergammaglobulinemia we observe in the plasma in stage 3 is partly due to antigen overload and hyper activation of the B cells which could occur in the extrafollicular areas of lymphoid tissues as damage to the lymph node has been shown to occur very early in acute infection [33]. This may play a greater role in driving B cell hyperactivation in stage 3. Overall, these results confirm a role for increased viremia in not only increasing plasma antibody responses but also mediating inflammation and skewing immune memory in acute HIV infection, resulting in impaired T cell- B cell interactions.

Resting memory B cells play a critical role in eliciting strong humoral immunity and were significantly lower in stage 3 than in stage 1/2. Interestingly, the frequencies of these resting memory B cells in both stage 1/2 and stage 3, but not chronic individuals were restored to levels similar to what is observed in healthy subjects. These results strongly suggest that intervention at the earliest stages of infection can maintain the integrity and survival of resting memory B cells, which are significantly capable of strengthening humoral defenses and very important in preventing HIV disease progression. This is similar to other studies comparing the effects of early ART versus initiation at the chronic phase, where the resting memory B cell subset was better restored with early treatment [44, 45].

As we investigate what occurs in the periphery, we are cognizant that this environment may not necessarily represent what occurs in GCs in secondary lymphoid tissue. Using cTfh and memory B cells as surrogates allows us to interpret what may be going on generally and these results will help is to refine our future investigations. However, based on our previous work [6, 24] and the results in this study, we propose the notion that cTfh-mediated B cell interaction in early acute infection is compromised in an inflammatory microenvironment. The exact mechanism of dysregulation is still not entirely clear, as both cell types appear to be affected during acute infection, so further investigation is needed. However, the idea to initiate ART very early in acute infection is validated as we observe that initiation of ART in the first week of infection leads to an environment with less inflammation. We hypothesize that this may prevent irreversible deleterious changes to the immune system, and may preserve immune function.

Of note, there has been pioneering but not extensive work done on the timing of ART for controlling viral rebound after treatment interruption. This study as well as work done by others, has shown that the timing of ART is important to preserve T cell-dependent B cell responses [44, 45] and mucosal T cell responses [46], all of which provide more insight into how preservation of memory could be important in overcoming viral rebound after ART interruption. Other studies have concluded there is no evidence for post-treatment control after ART interruption, even in the earliest participant group investigated (8.6 weeks from the estimated time of infection) [47]. Their observations however support what we believe occurs in acute HIV infection, and highlights the significant differences between participants our studies, in that initiating ART at 8.6 weeks from the estimated time of HIV infection is too late to preserve memory and hence prevent viral rebound after treatment interruption. As we were able to show in our current study, there is preservation of humoral function and memory in individuals that started ART from as early as 12 days from the history of exposure, with significant perturbations observed in individuals starting treatment at 18 days from the history of exposure. We therefore conclude that initiating ART at the earliest stages of HIV infection is key to preserving immune memory, and could prove most effective in controlling viral replication after treatment interruption.

## Materials and Methods

### Study participants

The RV254/SEARCH 010 study is an ongoing study in Bangkok, Thailand (clinicaltrials.gov NCT00796146), recruiting participants at the earliest stages of acute HIV infection. Peripheral blood mononuclear cells (PBMCs) from participants in the study cohort were used for these experiments (S1 and S2 Tables). Individuals with non-reactive HIV IgG were enrolled as previously described [48, 49], and later reclassified into stages 1, 2 or 3 using the 4th generation immunoassay (4thG IA) staging system [27]. Definitions for the stages were stage 1 (HIV RNA+, 4thG IA-, 3rd generation (3rdG) IA-, stage 2 (HIV RNA+, 4thG IA+, 3rdG IA-), and stage 3 (HIV RNA+, 4thG IA+, 3rdG IA+, Western Blot negative or indeterminate). Participants subsequently initiated ART at a median (IQR) of 2 (1–2) days from enrollment. Individuals from stage 1 and stage 2 were grouped together to represent stage 1/2, and compared to stage 3; as evaluating circulating Tfh function indicated that stage 2 clustered more similarly with stage 1 in the context of B cell help compared to stage 3. PBMCs from chronically HIV-infected (CHI) and HIV negative (HIV-) participants were also obtained for analysis from other protocols (SEARCH 011 and RV304/SEARCH 013 respectively) (S3 and S4 Tables).

### Ethics statement

The RV254/SEARCH010 study (NCT00796146) was approved by the Institutional Review Boards (IRBs) of Chulalongkorn University, Walter Reed Army Institute of Research (WRAIR), University of California at San Francisco (UCSF), Yale University, University of Hawaii (UH), University of Texas Medical Branch at Galveston, University of Sydney, and Centre Hospitalier de l’Université de Montréal Le Comité d’Ethique de la Recherche. The RV304/SEARCH013 study (NCT01397669) was approved by the IRBs of Chulalongkorn University, Walter Reed Army Institute of Research (WRAIR), University of California at San Francisco (UCSF), and Yale University. The SEARCH011 study (NCT00782808) was approved by the IRBs of Chulalongkorn University, University of California at San Francisco (UCSF), and University of Hawaii (UH). Initiation of ART was voluntary and done as part of enrollment. All participants gave written informed consent for all the studies detailed above.

### Coculture assay

PBMCs from HIV-infected patients were incubated with fluorochrome-conjugated antibodies for at least 15–20 minutes at 4°C or on ice, protected from light. Samples were sorted on a BD™ FACSAria II for: cTfh cells defined as CD3^+^CD4^+^CD45RA^-^CXCR5^+^CXCR3^-^, less efficient helper T cells denoted CXCR5^+^CXCR3^+^ and defined as CD3^+^CD4^+^CD45RA^-^CXCR5^+^CXCR3^+^; and resting memory B cells defined as CD3^-^CD19^+^CD10^-^CD27^+^. Sorted cTfh or CXCR5^+^CXCR3^+^ T cells were placed in coculture with sorted memory B cells at an equal ratio with 100ng/mL of staphylococcal enterotoxin B (SEB) (Toxin Technology) in RPMI 1640 (Corning) with 10% fetal bovine serum (Access Biologicals) and 1% penicillin/streptomycin (Gibco). SEB was required to induce T cell activation and promote T/B crosstalk [21]. Of note, total IgG measured in T-B cultures without SEB had very low levels (S2 Fig). Supernatant and cells were harvested at day 7 for subsequent analysis. The absolute number of cells were counted by flow cytometry with Flow-count Fluorospheres (Beckman Coulter) using the protocol recommended by the manufacturer.

### Flow cytometry and antibodies

PBMCs from HIV-infected or HIV-negative patients were incubated with fluorochrome-conjugated antibodies for at least 15–20 minutes at 4°C or on ice, protected from light. The following fluorochrome-conjugated anti-human antibodies were used: CD3 (HIT3α), CD4 (RPA-T4), CD10 (HI10a), CD20 (2H7), CD25 (BC96), CD27 (O323), CD38 (HIT2), PD-1 (EH12.2H7), and CXCR3 (G025H7) were all from BioLegend. CD19 (HIB19), CXCR5 (RF8 B2), ICOS (DX29) and CD21 (B-ly4) were from BD Biosciences and CD45RA (2H4LDH11LDB9) from Beckman Coulter. LIVE/DEAD Fixable Dead Cell Stain (Life Technologies) was used to gate on live cells; and in some cases both LIVE/DEAD stain and Annexin V (BD Biosciences) were used. Samples were acquired on a BD LSR II.

### Total and HIV-specific IgG ELISA

Total and HIV-specific IgG was measured by ELISA on culture supernatant and plasma as previously described [6]. Total IgG was detected by coating 96-well Immulon 2HB plates (Thermo Fisher Scientific) with anti-human monoclonal IgG (Mabtech, clone MT91/145) at a concentration of 1μg/mL in phosphate buffered saline (PBS) overnight at 4°C. The next day plates were washed 5 times (unless otherwise stated) with wash buffer (PBS + 0.05% Tween 20), and subsequently left to block with wash buffer for 1 hour at room temperature. Plates were then washed before the addition of sample and IgG standards at different dilutions, for 1 hour at room temperature. HIV Env-specific antibody responses were detected by coating 96-well high-binding half-area plates (Greiner Bio-One) with 1μg/mL recombinant HIV-1 envelope protein (ProSpec) in PBS and incubating overnight at 4°C. The next day plates were washed 5 times (unless otherwise stated) with wash buffer (PBS + 0.05% Tween 20), and subsequently left to block with wash buffer for 1 hour at room temperature. Plates were then washed before the addition of samples at different dilutions for 2 hours at room temperature. For the standard curve, human HIV immunoglobulin was used, and was obtained through the NIH AIDS Research and Reference Reagent Program, Division of AIDS, NIAD, NIH (Cat no. 3957) via Dr. Luiz Barbosa, NABI and National Heart, Lung and Blood Institute. For both assays, following sample incubation and washing, the plates were left to incubate with 1μg/mL of anti-human IgG-biotin (Mabtech, clone MT78/145) for 1 hour at room temperature. The wash step was repeated and the plates incubated with streptavidin-HRP (Mabtech) for 1 hour at room temperature. An extra wash was added to the last wash step before adding 100μL of TMB substrate (Sigma-Aldrich) to each well until a color change was observed. The reaction was stopped by the addition of 50μL of 1M H_3_PO_4_. The OD values were read at 450nm using a spectrophotometer (SpectraMax Plus, Molecular Devices).

### Multiplex cytokine assay

IL-1β, IL-6, IL-10, IFN-γ, MIP-1α, RANTES and TNF-α were measured in 7 day culture supernatant from T and B cell coculture experiments using the Bio-Plex Pro™ Human Cytokine Luminex Kit (Bio-Rad) exactly according the manufacturer’s instructions. Samples were added neat to the plate and cytokine standards were supplied by the manufacturer. The samples were acquired using a Bioplex-200 system and the data analyzed on the BioPlex Manager Software (Bio-Rad).

### Statistics

All data were analyzed using Graphpad Prism v10. Unpaired Student’s t test (Mann Whitney) was used when comparing two groups. The unpaired multiple t test and non-parametric one-way ANOVA (Dunn’s) test was used when comparing more than two groups to each other. Comparisons by correlations were analyzed using nonparametric spearman correlation. P values of less than 0.05 were considered statistically significant.

## Supporting Information

S1 Fig Impact of acute HIV infection on the cTfh population. PBMCs from Stage 1 and 2 and Stage 3 patients at week 0 (W0) (n = 17 and n = 16 respectively) were analyzed by flow cytometry. (A) Representative plots showing cTfh identified as CD3^+^CD4^+^CD45RA^-^CXCR5^+^CXCR3^-^, the less efficient helper T cells identified as CD3^+^CD4^+^CD45RA^-^CXCR5^+^CXCR3^+^; and non- helper T cells (denoted CXCR5^+^CXCR3^+^) identified as CD3^+^CD4^+^CD45RA^-^CXCR5^-^ cells. Frequencies of (B) cTfh and the frequency of (C) PD-1, (D) ICOS, (E) CD25 and (F) CD38 expression on cTfh was determined. (G) The mean fluorescence intensity (MFI) of CD38 was determined for cTfh after stimulation with SEB for 3 days. For graphs bars represent mean ±SD and symbols on the graphs represent stage 1/2 individuals (black circles) and stage 3 individuals (black squares). Statistics were carried out using the Mann-Whitney non-parametric test. * P< 0.05.
(TIF)

S2 Fig Different CXCR5^+^ helper T cell populations can give help to B cells at varying degrees. PBMCs from week 0 stage 1/2 (n = 9) and stage 3 (n = 4–7) individuals were sorted. cTfh cells (CXCR5^+^CXCR3^-^), (CXCR5^+^CXCR3^+^) were placed in culture with autologous CD10^-^CD21^+^CD27^+^ resting memory B cells in the presence of or without SEB. Quantification of cTfh-mediated B cell help was carried out by measuring total IgG ELISA in 7 day culture supernatant.
(TIF)

S3 Fig cTfh-B functional cytokine profile. Cocultures of cTfh and autologous resting memory B cells from week 0 stage 1/2 (n = 9) and stage 3 (n = 7) individuals were analyzed for the presence of cytokines (A) IL-1β, (B) IL-6, (C) IFN-γ and (D) MIP-1^α^. Bars represent mean ±SD. Symbols on the graphs represent stage 1/2 individuals (black circles) and stage 3 individuals (black squares) and statistics were carried out using the Mann-Whitney non-parametric test.
(TIF)

S4 Fig Gating strategies for mature B cell populations. (A) Terminally differentiated plasmablasts in stage 1/2 and stage 3 individuals from week 0 were identified as CD19^+^CD10^-^CD20^-^CD21^-^CD38^hi^CD27^hi^ cells. (B) Representative plots from a healthy individual showing total B cells identified as CD19^+^; activated memory (AM) B cells CD19^+^CD10^-^CD21^-^CD27^+^ cells, Tissue-like (TL) B cells CD19^+^CD10^-^CD21^-^CD27^-^, resting memory (RM) B cells CD19^+^CD10^-^CD21^+^CD27^+^ and resting naïve (RN) B cells CD19^+^CD10^-^CD21^+^CD27^-^IgD^+^.
(TIF)

S5 Fig Restoration of functionally impaired cTfh-mediated B cell response is possible. cTfh cells from a sorted pool of healthy controls (HC) were used to substitute cTfh cells from stage 3 HIV-infected individuals placed in coculture with memory B cells from stage 3 subjects. (A) Total IgG, (B) HIV-specific IgG, (C) IL-10 and (D) RANTES levels were assessed in coculture supernatant. Symbols on the graphs represent cTfh from Stage 3 individuals (black squares) and cTfh from HCs (open diamonds). Statistics carried out using the Mann-Whitney non-parametric test.
(TIF)

S6 Fig Functionality of resting memory B cells. CFSE labeled sorted CD21^+^CD27^+^ resting memory B cells from stage 1/2 and stage 3 (n = 4–5) were stimulated in vitro with CpG ODN for 5 days. (A) CFSE expression on cells was analyzed by flow cytometry and (B) total IgG was quantified in the supernatant by ELISA. (C) The expression of BCL2 on CD21^+^CD27^+^ memory B cells from stage 1/2 and stage 3 individuals from day 0 was measured ex vivo by flow cytometry. Symbols on the graphs represent stage 1/2 individuals (black circles) and stage 3 individuals (black squares). Statistics were carried out using the Mann-Whitney non-parametric test. * P< 0.05.
(TIF)

S1 Table Participant Information on the 4^th^G stage 1and 2 acute HIV-infected individuals used in assays. (DOCX)

S2 Table Participant Information on the 4^th^G stage 3 acute HIV-infected individuals used in assays. (DOCX)

S3 Table Participant Information on chronic HIV-infected individuals used in assays. (DOCX)

S4 Table Participant Information on HIV negative individuals used in assays. (DOCX)
