# Supplementary figures and images for: Altered Memory Circulating T Follicular Helper-B Cell Interaction in Early Acute HIV Infection
Source: PLoS Pathog. 2016 Jul 27;12(7):e1005777. doi: 10.1371/journal.ppat.1005777 (PMC4963136; doi:10.1371/journal.ppat.1005777)

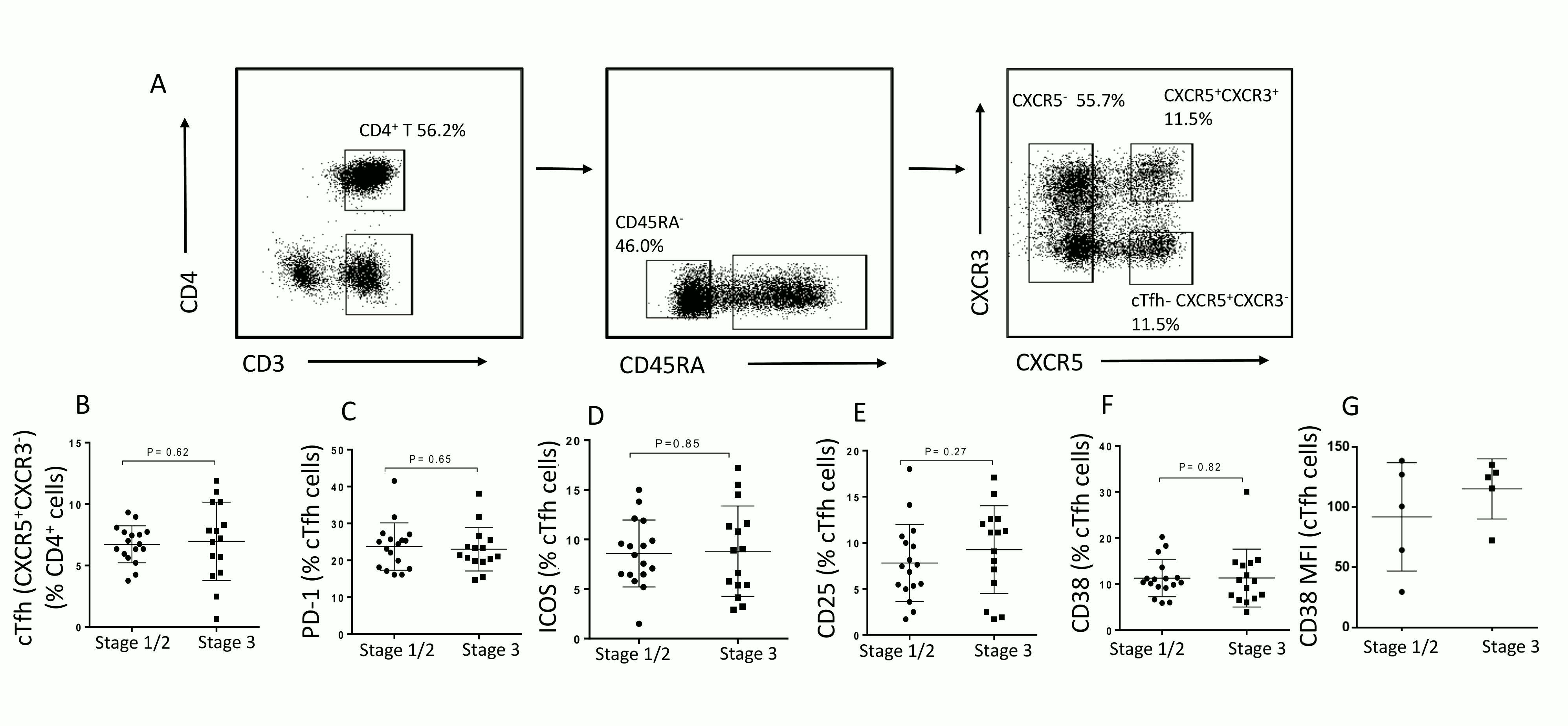

Supplement: S1 Fig — PBMCs from Stage 1 and 2 and Stage 3 patients at week 0 (W0) (n = 17 and n = 16 respectively) were analyzed by flow cytometry. (A) Representative plots showing cTfh identified as CD3+CD4+CD45RA-CXCR5+CXCR3-, the less efficient helper T cells identified as CD3+CD4+CD45RA-CXCR5+CXCR3+; and non- helper T cells (denoted CXCR5+CXCR3+) identified as CD3+CD4+CD45RA-CXCR5- cells. Frequencies of (B) cTfh and the frequency of (C) PD-1, (D) ICOS, (E) CD25 and (F) CD38 expression on cTfh was determined. (G) The mean fluorescence intensity (MFI) of CD38 was determined for cTfh after stimulation with SEB for 3 days. For graphs bars represent mean ±SD and symbols on the graphs represent stage 1/2 individuals (black circles) and stage 3 individuals (black squares). Statistics were carried out using the Mann-Whitney non-parametric test. * P< 0.05. (TIF) [file ppat.1005777.s001.tif]

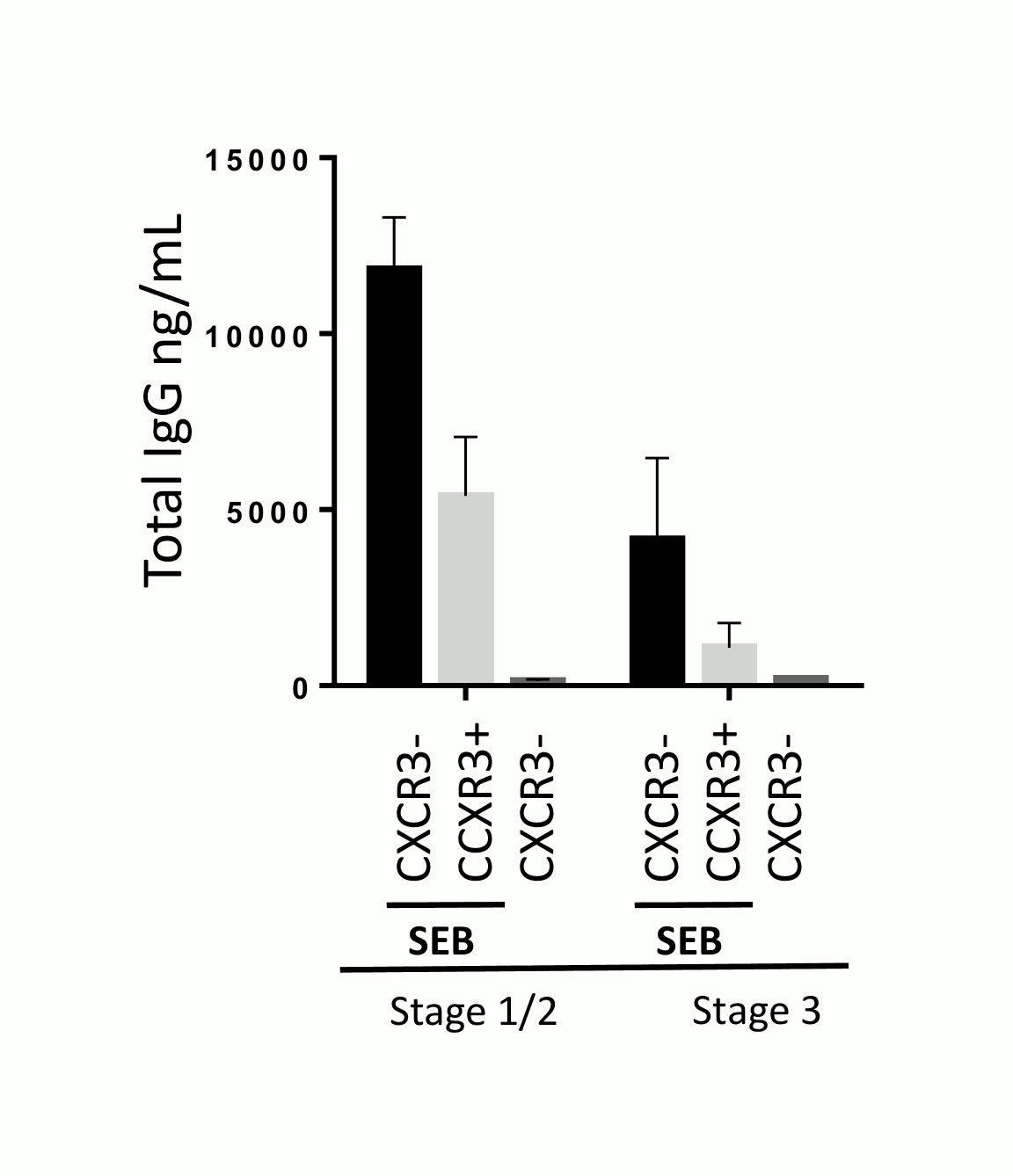

Supplement: S2 Fig — PBMCs from week 0 stage 1/2 (n = 9) and stage 3 (n = 4–7) individuals were sorted. cTfh cells (CXCR5+CXCR3-), (CXCR5+CXCR3+) were placed in culture with autologous CD10-CD21+CD27+ resting memory B cells in the presence of or without SEB. Quantification of cTfh-mediated B cell help was carried out by measuring total IgG ELISA in 7 day culture supernatant. (TIF) [file ppat.1005777.s002.tif]

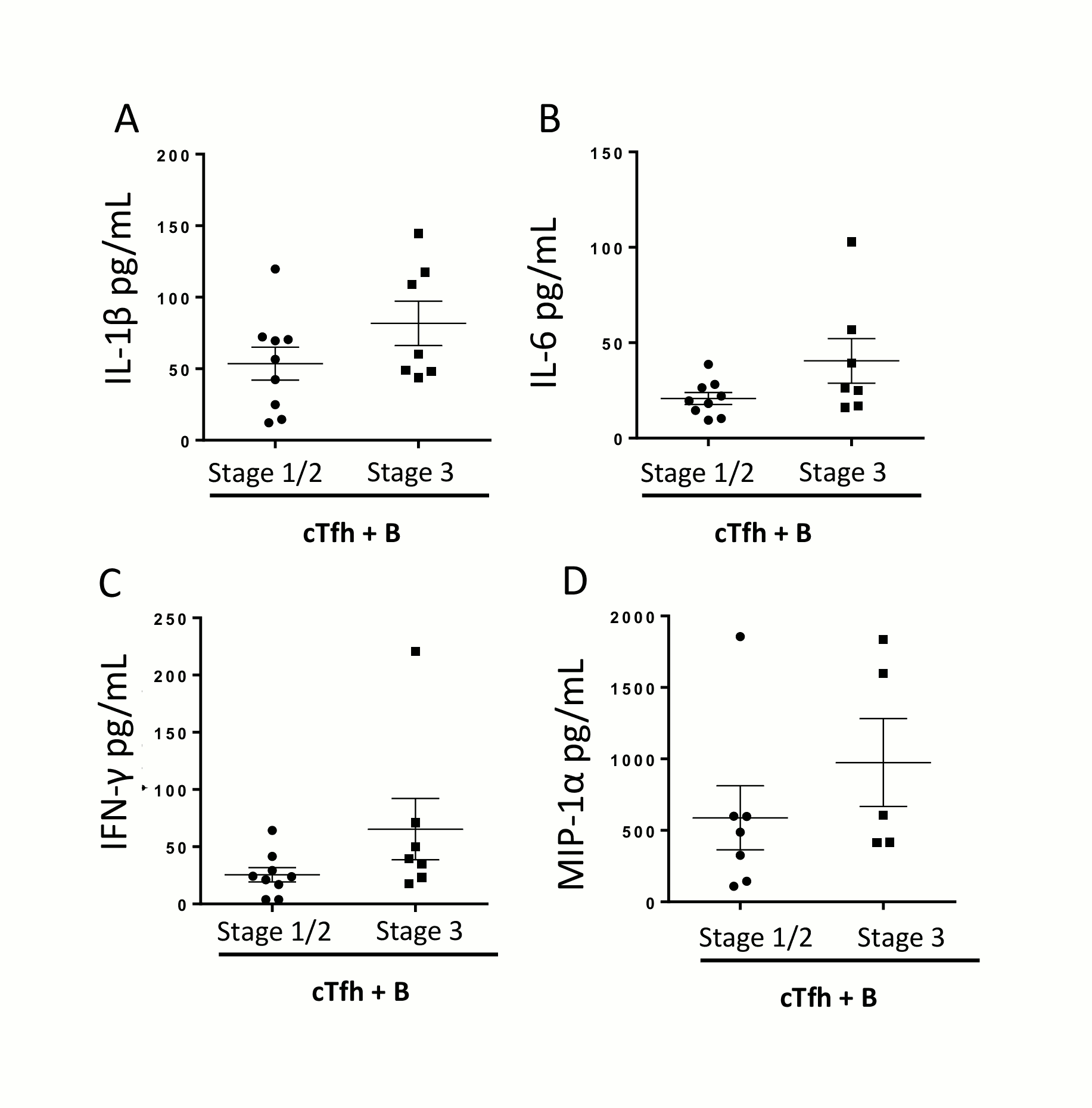

Supplement: S3 Fig — Cocultures of cTfh and autologous resting memory B cells from week 0 stage 1/2 (n = 9) and stage 3 (n = 7) individuals were analyzed for the presence of cytokines (A) IL-1β, (B) IL-6, (C) IFN-γ and (D) MIP-1α. Bars represent mean ±SD. Symbols on the graphs represent stage 1/2 individuals (black circles) and stage 3 individuals (black squares) and statistics were carried out using the Mann-Whitney non-parametric test. (TIF) [file ppat.1005777.s003.tif]

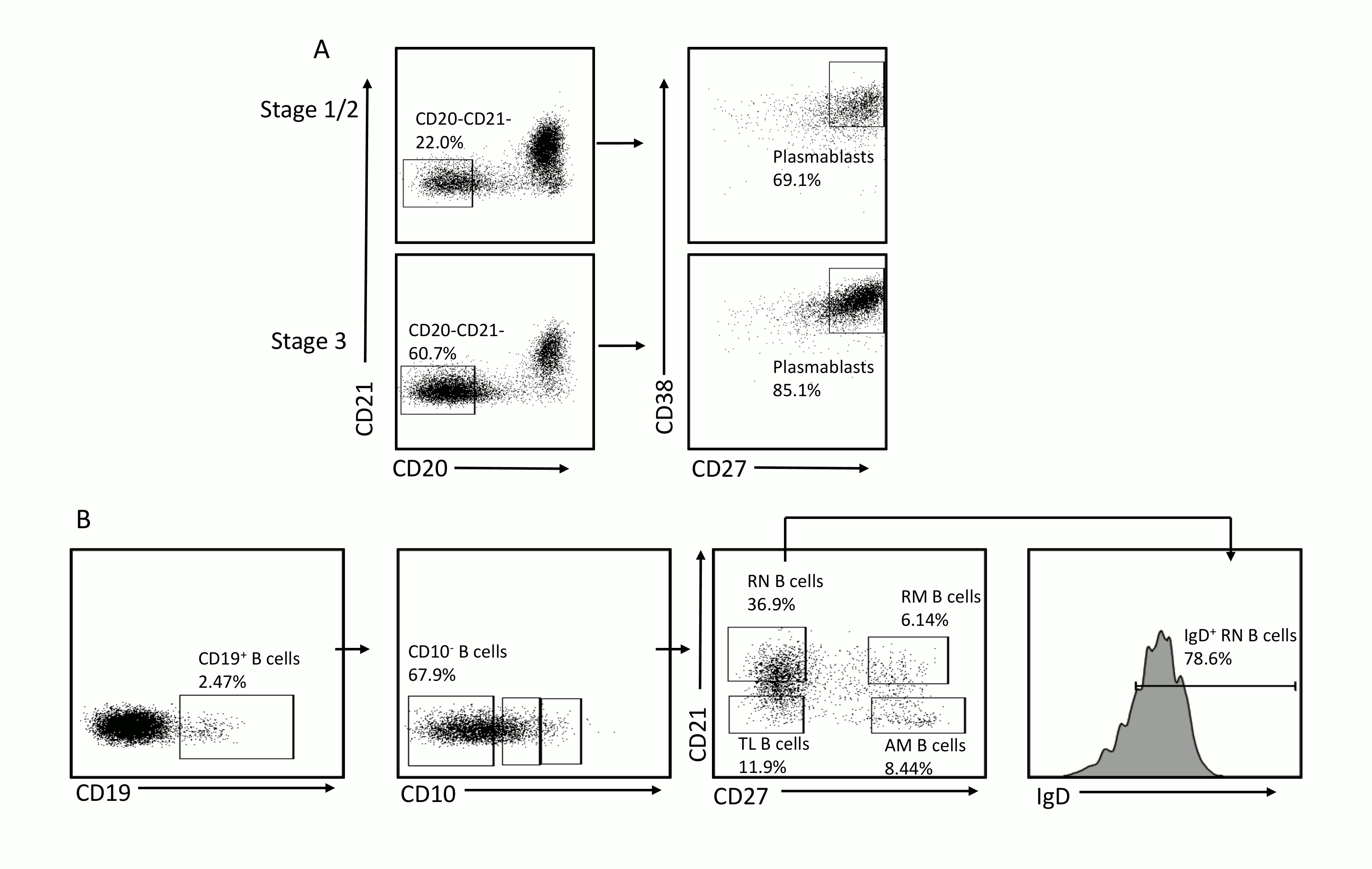

Supplement: S4 Fig — (A) Terminally differentiated plasmablasts in stage 1/2 and stage 3 individuals from week 0 were identified as CD19+CD10-CD20-CD21-CD38hiCD27hi cells. (B) Representative plots from a healthy individual showing total B cells identified as CD19+; activated memory (AM) B cells CD19+CD10-CD21-CD27+ cells, Tissue-like (TL) B cells CD19+CD10-CD21-CD27-, resting memory (RM) B cells CD19+CD10-CD21+CD27+ and resting naïve (RN) B cells CD19+CD10-CD21+CD27-IgD+. (TIF) [file ppat.1005777.s004.tif]

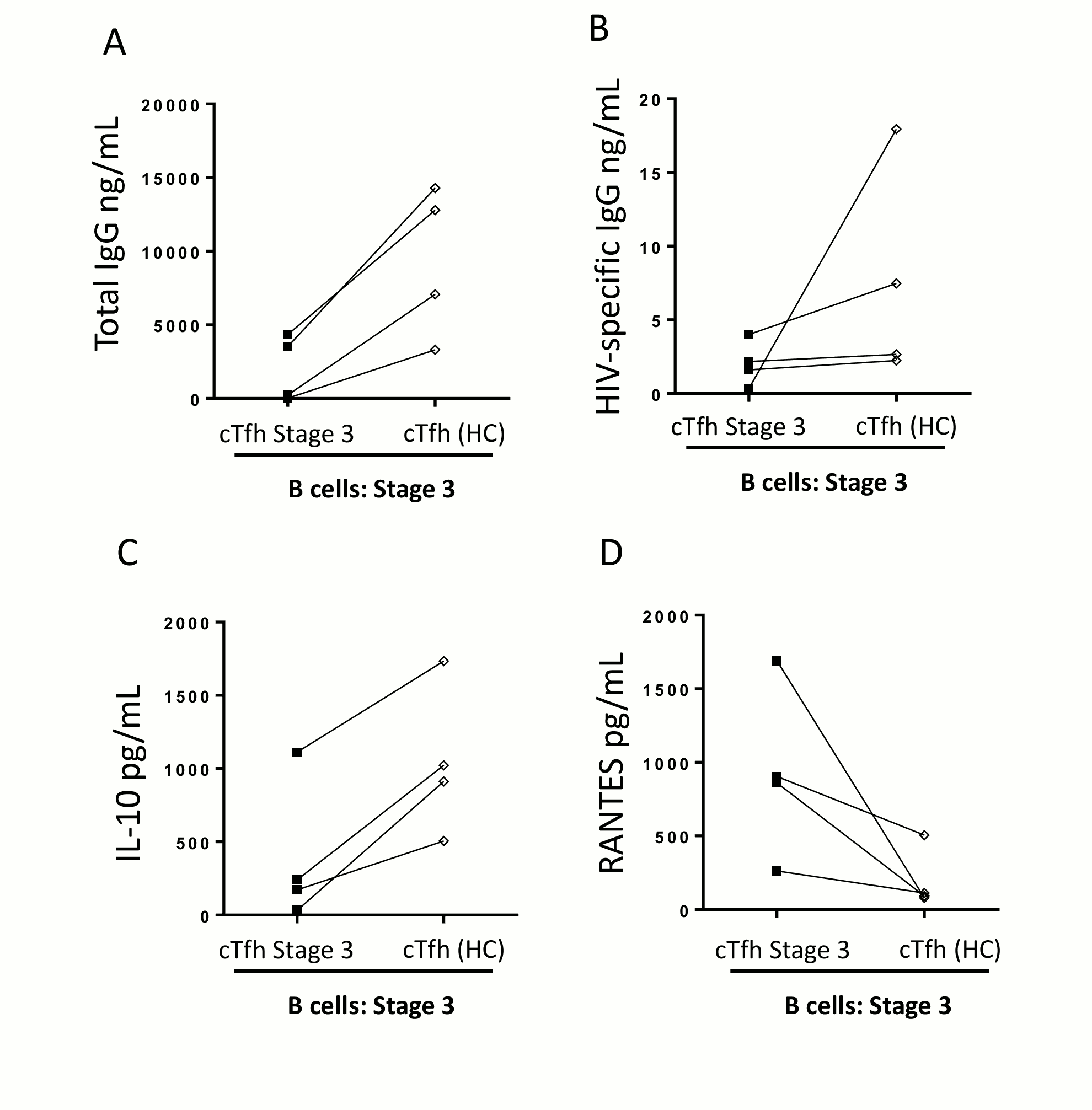

Supplement: S5 Fig — cTfh cells from a sorted pool of healthy controls (HC) were used to substitute cTfh cells from stage 3 HIV-infected individuals placed in coculture with memory B cells from stage 3 subjects. (A) Total IgG, (B) HIV-specific IgG, (C) IL-10 and (D) RANTES levels were assessed in coculture supernatant. Symbols on the graphs represent cTfh from Stage 3 individuals (black squares) and cTfh from HCs (open diamonds). Statistics carried out using the Mann-Whitney non-parametric test. (TIF) [file ppat.1005777.s005.tif]

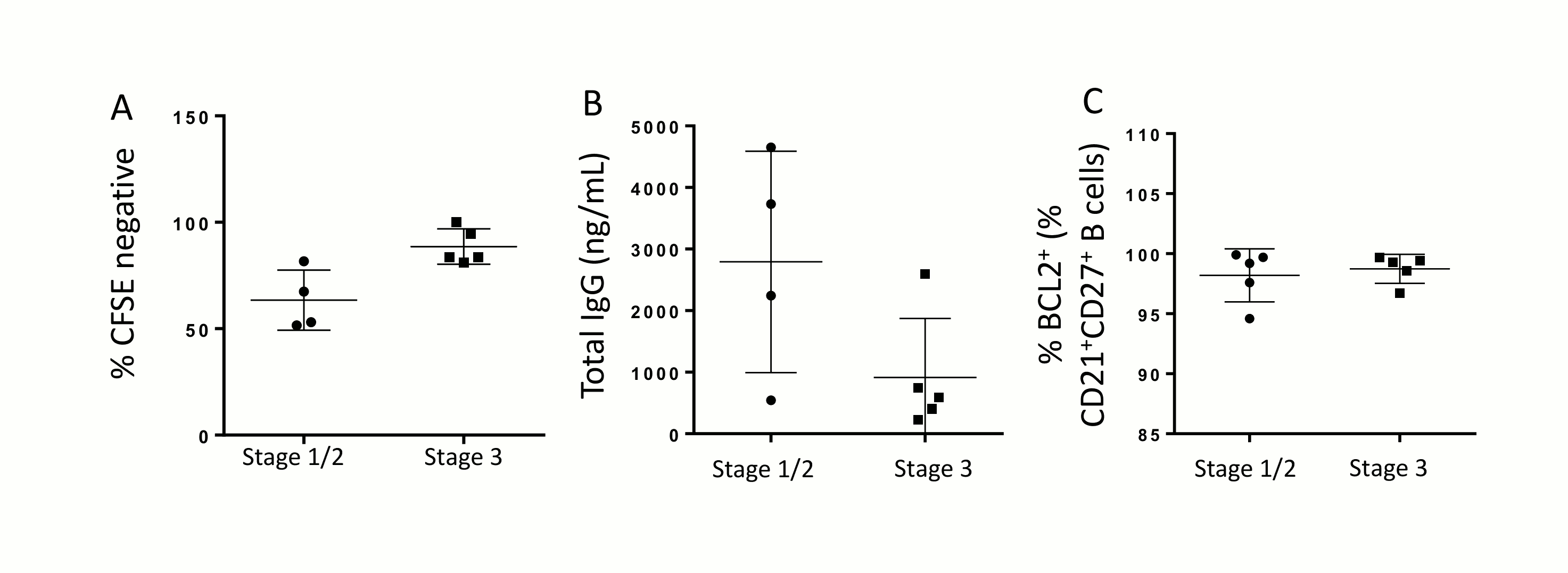

Supplement: S6 Fig — CFSE labeled sorted CD21+CD27+ resting memory B cells from stage 1/2 and stage 3 (n = 4–5) were stimulated in vitro with CpG ODN for 5 days. (A) CFSE expression on cells was analyzed by flow cytometry and (B) total IgG was quantified in the supernatant by ELISA. (C) The expression of BCL2 on CD21+CD27+ memory B cells from stage 1/2 and stage 3 individuals from day 0 was measured ex vivo by flow cytometry. Symbols on the graphs represent stage 1/2 individuals (black circles) and stage 3 individuals (black squares). Statistics were carried out using the Mann-Whitney non-parametric test. * P< 0.05. (TIF) [file ppat.1005777.s006.tif]
