# Supplementary material for: Altered Memory Circulating T Follicular Helper-B Cell Interaction in Early Acute HIV Infection
Source: PLoS Pathog. 2016 Jul 27;12(7):e1005777. doi: 10.1371/journal.ppat.1005777 (PMC4963136; doi:10.1371/journal.ppat.1005777)
Supplement: S1 Table — (DOCX) [file ppat.1005777.s007.docx]

| **Patient ID** | **Cohort** | **Age** | **Gender** | **4thG Stage** | **Plasma HIV RNA** | **W0/V1 CD4+ T cell count** | **W0/V1 CD8+ T cell count** | **W72^ƚ^/V17 CD4+ T cell count** | **W72^ƚ^/V17 CD8+ T cell count** |
| --- | --- | --- | --- | --- | --- | --- | --- | --- | --- |
|  |  |  |  |  | (copies/ml) | ( cells/µl) | (cells/µl) | (cells/µl) | (cells/µl) |
| 1A | SEARCH010 | 28 | M | 1 | 276,124 | 311 | 271 | 724 | 450 |
| 2A | SEARCH010 | 41 | M | 1 | 2,321 | 547 | 348 | 543 | 292 |
| 3A | SEARCH010 | 22 | M | 2 | 71,472 | 257 | 213 | 654 | 362 |
| 4A | SEARCH010 | 31 | F | 1 | 3,897 | 970 | 586 | 1224 | 808 |
| 5A | SEARCH010 | 42 | M | 1 | 4,452 | 641 | 442 | 593 | 389 |
| 6A | SEARCH010 | 28 | M | 1 | 280,700 | 249 | 198 | 702 | 626 |
| 7A | SEARCH010 | 39 | M | 2 | 249,679 | 413 | 135 | 1021 | 346 |
| 8A | SEARCH010 | 25 | M | 2 | 54,000 | 618 | 448 | 591 | 463 |
| 9A | SEARCH010 | 26 | F | 2 | 662,035 | 342 | 201 | 904 | 732 |
| 10A | SEARCH010 | 46 | M | 1 | 31,970 | 447 | 399 | 543 | 305 |
| 11A | SEARCH010 | 23 | M | 2 | 708,317 | 265 | 160 | NA | NA |
| 12A | SEARCH010 | 24 | M | 1 | 11,494 | 565 | 879 | 624 | 1022 |
| 13A | SEARCH010 | 18 | M | 1 | 1,089 | 371 | 412 | 429 | 405 |
| 14A | SEARCH010 | 22 | M | 1 | 322,660 | 320 | 205 | 563 | 688 |
| 15A | SEARCH010 | 29 | M | 2 | 7,263,860 | 213 | 127 | 507 | 384 |
| 16A | SEARCH010 | 34 | M | 2 | 237,077 | 362 | 299 | 911 | 623 |
| 17A | SEARCH010 | 34 | M | 2 | 470,496 | 214 | 214 | 726 | 726 |
| 18A | SEARCH010 | 37 | M | 2 | 121,377 | 698 | 224 | 994 | 454 |
| 19A | SEARCH010 | 34 | M | 2 | 1,170,480 | 265 | 240 | 473 | 728 |
| 20A | SEARCH010 | 48 | M | 1 | 268,663 | 538 | 238 | 598 | 279 |
| 21A | SEARCH010 | 44 | M | 2 | 426027 | 303 | 204 | 788 | 557 |
| 22A | SEARCH010 | 28 | M | 1 | 1044 | 1236 | 843 | 1424 | 1499 |
| 23A | SEARCH010 | 24 | M | 1 | 85265 | 261 | 447 | 673 | 1045 |
| 24A | SEARCH010 | 31 | M | 2 | 262366 | 395 | 232 | 815 | 510 |
| 25A | SEARCH010 | 30 | M | 1 | 41999 | 227 | 279 | 452 | 437 |
| 26A | SEARCH010 | 24 | M | 2 | 58434 | 457 | 343 | NA | NA |

^Ƚ^ W72- 72 weeks of antiretroviral treatment

NA- not available
