# Supplementary material for: Altered Memory Circulating T Follicular Helper-B Cell Interaction in Early Acute HIV Infection
Source: PLoS Pathog. 2016 Jul 27;12(7):e1005777. doi: 10.1371/journal.ppat.1005777 (PMC4963136; doi:10.1371/journal.ppat.1005777)
Supplement: S2 Table — (DOCX) [file ppat.1005777.s008.docx]

| **Patient ID** | **Cohort** | **Age** | **Gender** | **4thG Stage** | **Plasma HIV RNA** | **W0/V1 CD4+ T cell count** | **W0/V1 CD8+ T cell count** | **W72^ƚ^/V17 CD4+ T cell count** | **W72^ƚ^/V17 CD8+ T cell count** |
| --- | --- | --- | --- | --- | --- | --- | --- | --- | --- |
|  |  |  |  |  | **(copies/ml)** | **( cells/µl)** | **(cells/µl)** | **(cells/µl)** | **(cells/µl)** |
| 27A | SEARCH010 | 28 | F | 3 | 7,354,810 | 341 | 264 | 610 | 337 |
| 28A | SEARCH010 | 29 | F | 3 | 571,082 | 463 | 1227 | NA | NA |
| 29A | SEARCH010 | 30 | M | 3 | 388,339 | 293 | 518 | 462 | 296 |
| 30A | SEARCH010 | 33 | M | 3 | 358,198 | 386 | 570 | 480 | 392 |
| 31A | SEARCH010 | 45 | F | 3 | 25,579,700 | 132 | 425 | 490 | 556 |
| 32A | SEARCH010 | 25 | M | 3 | 1,642,350 | 298 | 638 | 479 | 346 |
| 33A | SEARCH010 | 28 | M | 3 | 277,036 | 389 | 1058 | 558 | 581 |
| 34A | SEARCH010 | 22 | M | 3 | 624,066 | 496 | 690 | 821 | 718 |
| 35A | SEARCH010 | 37 | M | 3 | 14,240,700 | 198 | 402 | 594 | 914 |
| 36A | SEARCH010 | 29 | M | 3 | 61,858 | 352 | 405 | 585 | 488 |
| 37A | SEARCH010 | 28 | M | 3 | 13,557,900 | 359 | 497 | 596 | 398 |
| 38A | SEARCH010 | 20 | M | 3 | 2,202,820 | 532 | 510 | 960 | 1013 |
| 39A | SEARCH010 | 34 | M | 3 | 17,811,300 | 292 | 528 | 591 | 608 |
| 40A | SEARCH010 | 22 | M | 3 | 2,656,900 | 350 | 672 | 498 | 936 |
| 41A | SEARCH010 | 32 | M | 3 | 152,807 | 466 | 316 | 926 | 805 |
| 42A | SEARCH010 | 23 | M | 3 | 47,887,300 | 392 | 228 | 480 | 320 |
| 43A | SEARCH010 | 25 | M | 3 | 8,001,600 | 210 | 216 | 727 | 512 |
| 44A | SEARCH010 | 27 | M | 3 | 22,516,400 | 182 | 509 | 448 | 480 |
| 45A | SEARCH010 | 20 | M | 3 | 77,893 | 354 | 549 | 586 | 566 |
| 46A | SEARCH010 | 29 | M | 3 | >10000000 | 152 | 251 | 451 | 846 |
| 47A | SEARCH010 | 27 | M | 3 | 7,249,767 | 234 | 652 | NA | NA |

^Ƚ^ W72- 72 weeks of antiretroviral treatment

NA- not available
