# Supplementary material for: Altered Memory Circulating T Follicular Helper-B Cell Interaction in Early Acute HIV Infection
Source: PLoS Pathog. 2016 Jul 27;12(7):e1005777. doi: 10.1371/journal.ppat.1005777 (PMC4963136; doi:10.1371/journal.ppat.1005777)
Supplement: S3 Table — (DOCX) [file ppat.1005777.s009.docx]

| **Patient ID** | **Cohort** | **Age** | **Gender** | **Stage** | **Plasma HIV RNA** | **CD4+ T cell count** | **CD8+ T cell count** |
| --- | --- | --- | --- | --- | --- | --- | --- |
|  |  |  |  |  | **(copies/ml)** | **( cells/µl)** | **(cells/µl)** |
| 1C | SEARCH011 | 37 | M | CHI | 350,439 | 30 | 936 |
| 2C | SEARCH011 | 38 | M | CHI | 98,808 | 239 | 1030 |
| 3C | SEARCH011 | 31 | M | CHI | 750,000 | 192 | 691 |
| 4C | SEARCH011 | 33 | M | CHI | 111,344 | 114 | 1705 |
| 5C | SEARCH011 | 48 | M | CHI | 750,000 | 52 | 464 |
| 6C | SEARCH011 | 42 | M | CHI | 40,227 | 360 | 1433 |
| 7C | SEARCH011 | 34 | F | CHI | 47,679 | 235 | 587 |
| 8C | SEARCH011 | 24 | F | CHI | 91,619 | 284 | 1245 |
| 9C | SEARCH011 | 35 | F | CHI | 49,553 | 178 | 397 |
| 10C | SEARCH011 | 46 | M | CHI | 58,320 | 386 | 987 |
| 11C | SEARCH011 | 29 | F | CHI | 484,000 | 219 | 641 |
| 12C | SEARCH011 | 34 | M | CHI | 1,398 | 456 | 1064 |
| 13C | SEARCH011 | 45 | M | CHI | 41,626 | 136 | 1180 |
| 14C | SEARCH011 | 31 | F | CHI | 36,214 | 363 | 726 |
