# Supplementary material for: Altered Memory Circulating T Follicular Helper-B Cell Interaction in Early Acute HIV Infection
Source: PLoS Pathog. 2016 Jul 27;12(7):e1005777. doi: 10.1371/journal.ppat.1005777 (PMC4963136; doi:10.1371/journal.ppat.1005777)
Supplement: S4 Table — (DOCX) [file ppat.1005777.s010.docx]

| **Patient ID** | **Cohort** | **Age** | **Gender** | **Stage** |
| --- | --- | --- | --- | --- |
| 1N | SEARCH013 | 39 | M | HIV NEG |
| 2N | SEARCH013 | 29 | F | HIV NEG |
| 3N | SEARCH013 | 24 | F | HIV NEG |
| 4N | SEARCH013 | 39 | M | HIV NEG |
| 5N | SEARCH013 | 24 | M | HIV NEG |
| 6N | SEARCH013 | 32 | M | HIV NEG |
| 7N | SEARCH013 | 42 | M | HIV NEG |
| 8N | SEARCH013 | 32 | F | HIV NEG |
| 9N | SEARCH013 | 33 | F | HIV NEG |
| 10N | SEARCH013 | 36 | M | HIV NEG |
| 11N | SEARCH013 | 29 | M | HIV NEG |
| 12N | SEARCH013 | 34 | F | HIV NEG |
| 13N | SEARCH013 | 21 | M | HIV NEG |
| 14N | SEARCH013 | 44 | F | HIV NEG |
